# Screening and Analysis of Serum Protein Biomarkers Infected by Coronavirus Disease 2019 (COVID-19)

**DOI:** 10.3390/tropicalmed7120397

**Published:** 2022-11-25

**Authors:** Zhaomin Feng, Yang Pan, Yimeng Liu, Jiachen Zhao, Xiaomin Peng, Guilan Lu, Weixian Shi, Daitao Zhang, Shujuan Cui

**Affiliations:** Institute for Infectious Disease and Endemic Disease Control, Beijing Center for Disease Prevention and Control, Beijing 100013, China

**Keywords:** COVID-19, protein biomarkers, HPLC, LC-MS/MS

## Abstract

Coronavirus disease 2019 (COVID-19) has spread widely around the world, and in-depth research on COVID-19 is necessary for biomarkers and target drug discovery. This analysis collected serum from six COVID-19-infected patients and six healthy people. The protein changes in the infected and healthy control serum samples were evaluated by liquid chromatography-tandem mass spectrometry (LC-MS/MS) and high-performance liquid chromatography (HPLC). The differential protein signature in both groups was retrieved and analyzed by the Kyoto Encyclopedia of Gene and Genomes (KEGG), Gene ontology, COG/KOG, protein–protein interaction, and protein domain interactions tools. We shortlisted 24 differentially expressed proteins between both groups. Ten genes were significantly up-regulated in the infection group, and fourteen genes were significantly down-regulated. The GO and KEGG pathway enrichment analysis suggested that the chromosomal part and chromosome were the most enriched items. The oxytocin signaling pathway was the most enriched item of KEGG analysis. The netrin module (non-TIMP type) was the most enriched protein domain in this study. Functional analysis of S100A9, PIGR, C4B, IL-6R, IGLV3-19, IGLV3-1, and IGLV5-45 revealed that SARS-CoV-2 was closely related to immune response.

## 1. Introduction

As per the World Health Organization (WHO), COVID-19 is a rapidly spreading disease caused due to the infection of the 2019 new acute coronavirus (severe acute respiratory syndrome coronavirus-2, SARS-CoV-2) [1,2]. The continuous spread of this disease is the third coronavirus pandemic worldwide in the past two decades. SARS-CoV-2, SARS-CoV, and the Middle-East respiratory syndrome coronavirus (MERS-CoV) belong to the genus that caused the pandemic [3]. However, SARS-CoV-2 has unique infection characteristics, such as strong infectivity [4], long incubation period [5], general population susceptibility [6], no obvious infection symptoms in the early stage, and asymptomatic patients [7]. These features will be more conducive to the spread of SARS-CoV-2 among people. The basic regeneration number (R0) of SARS-CoV-2 is more than SARS-CoV and MERS-CoV without human intervention [8]. Furthermore, SARS-CoV-2 can be transmitted through close contact transmission, droplet transmission, fecal mouth transmission, and other methods [9,10]. Therefore, in-depth research on SARS-CoV-2 to identify the differentially regulated biomarkers and target proteins is essential.

The serum/plasma contains thousands of proteins produced by various tissues/cells of the body. Changes in the concentration, structure, and function of serum proteins suggest an abnormal pathophysiological state [11]. Therefore, the serum proteome study of patients infected with viruses helps discover the biomarkers of viral infections, establish early diagnostic methods, monitor disease progression, and predict therapeutic effects [12]. Proteomics technology is being widely used to study the interaction between viruses and human plasma. For example, Pang et al. reported two valuable SARS-CoV diagnostic markers: the complement C3c α chain N-terminal fragment and the fibrinogen α-E intra-chain fragment with specificity and sensitivity of over 95% [13]. Kang et al. employed four specific differentially expressed proteins (DEPs) in the serum of patients with acute SARS infection as variables to establish the diagnostic method of acute SARS proteome fingerprints using the decision tree classification algorithm. A double-blind study showed that the accuracy of the diagnostic method for identifying acute SARS and non-acute SARS samples was more than 95% [14]. Moreover, Wan et al. compared and analyzed the SARS case progression stage, recovery stage, and normal human plasma proteome [15]. The results indicated that SARS-CoV infection activates an excessive immune response, and enhanced inflammation may play a crucial role in the disease progression. Furthermore, the proteome of the plasma of patients infected with SARS at different onset times and the plasma of healthy participants was analyzed [16]. Quantitative proteomic methods can reveal the proteomic changes in the bronchoalveolar lavage fluid of critically ill patients with COVID-19, and help to screen out proteins that may be protein markers or therapeutic targets of COVID-19, thus providing new information for the research of anti-inflammatory drugs related to COVID-19 and the exploration of the molecular mechanism of the host response [17].

In this study, we have employed liquid chromatography-tandem mass spectrometry (LC-MS/MS) and high-performance liquid chromatography (HPLC) to probe the potential protein changes in the serum of the SARS-CoV-2 infected group compared with the healthy group. DEP and their functions were identified. The results obtained are helpful for the further study of the mechanisms of SARS-CoV-2 infection.

## 2. Materials and Methods

### 2.1. Serum Samples

The Ethics Committee of the Beijing Center for Disease Prevention and Control reviewed and approved all experimental protocols. The code is 2020031. Blood samples were collected from six COVID-19 patients and six healthy people, followed by serum isolation for further analysis. All patients signed the informed consent. The COVID-19 infection was diagnosed by the real-time RT-PCR recommended by China CDC in six male patients aged between 30 and 40 years. The six healthy people were also all male and aged between 30 and 40 years. All samples underwent liquid nitrogen snap freezing and were kept at −80 °C. All samples were initially centrifuged at 12,000× *g* for 10 min at 4 °C to be rid of the cellular debris. The PierceTM Top 12 Abundance protein Depletion spin columns kit was used to separate 12 highly abundant proteins from the supernatant after it had been transferred to a different centrifuge tube (Thermo Fisher, Waltham, MA, USA). Following the manufacturer’s instructions, the manufacturer’s BCA kit was used to measure the total protein. This study takes into account the high variability of characteristics caused by the SARS-CoV-2 virus in human beings and the homogeneity of the research object when screening subjects because it attempts to infer the generality from SARS-CoV-2 infected patients. Therefore, the severity of these six patients is the same; they are all mild patients, and they do not need oxygen treatment.

### 2.2. Trypsin Digestion

The protein sample was incubated with dithiothreitol (5 mM) at 56 °C for 30 min. Then, the protein sample was supplemented with iodoacetamide (11 mM) in the dark at room temperature for 15 min and lysed. Later, the samples were added with 100 mM TEAB, keeping the urea concentration below 2 M. Finally, trypsin was used for final digestion at two different mass ratios. First, digestion was performed at a 1:50 ratio overnight, followed by a 1:100 mass ratio for 4 h.

### 2.3. HPLC Analysis

A high pH reverse phase HPLC was used to extract the tryptic peptide fractions by the Agilent 300 extend C18 column (4.6 mm ID, 250 mm length, 5 μm particle). An initial 60 fractions of the peptides were extracted using an 8 to 32% gradient of acetonitrile (pH 9.0) in 60 min. The total fractions were combined into 18 fractions and vacuum dried through centrifugation.

### 2.4. LC-MS-MS Analysis

Tryptic peptides were dissolved in solvent A, which contains 0.1% formic acid. They were then loaded onto a reverse-phase analytical column. An increasing gradient was then made using solvent B, which contains 0.1% formic acid. The rising gradient was made up of a mixture of varying degrees of formic acid, starting from around 6 to 23% and going up to 80% in 3 min. Using an EASY-nLC system, the flow rate of 400 nL per minute was maintained. After the initial nanospray ionization process, the samples were subjected to multiple MS/MS tests using the Q ExactiveTM Plus platform from Thermo Scientific. The electrospray was set to 2.0 kV. The intifcation of the peptide was performed using an Orbitrap, which produced a resolution of 70,000 to 1800. The NCE setting was used to select the peptides for the MS/MS tests. Orbitrap was then used to achieve a resolution of 17,500. The data dependence procedure was performed on different scans with a dynamic exclusion of around 15 s. The fixed mass was then adjusted to 100 *m*/*z*.

### 2.5. Database Search

The data from the MS/MS were processed using Maxquant v. 1.52.8. Tandem MS data were thoroughly compared to the reverse decoy database and the SwissProt Human database. Trypsin/P was chosen as the cleavage enzyme for the two uncompleted cleavages. The mass tolerance of the precursor was set at 20 ppm for the initial search and was thereafter decreased to 5 ppm for the main search, with the mass tolerance of the fragments adjuted to 0.02 Da. The carbamidomethyl on the Cys residue was designated as a permanent modification, whereas the oxidation on the Met residue was designated as a variable modification. The minimum score was changed to >40, and the FDR value was set at 1%.

### 2.6. Gene Ontology Analysis

The gene ontology annotation (GOA) (https://www.ebi.ac.uk/GOA/index, accessed on 26 January 2021) program aims to provide annotations to protein databases in UniProt. Initially, the identified protein is converted into UniProt ID, followed by mapping to GO IDs. In the case of an unannotated protein ID, the InterProScan program was used to annotate the protein based on the protein function that was hypothesized by the sequence alignment approach. Then, according to GOA, these proteins are divided into three groups: cell component, biological process, and molecular function. A Fisher’s exact test (two-tailed) was implemented to examine the enrichment of the proteins in comparison to the identified proteins, and *p* < 0.05 was judged as significant.

### 2.7. Pathway Analysis

To predict the functional role of the discovered proteins, the Kyoto Encyclopedia of Genes and Genomes (KEGG) (https://www.genome.jp/kegg/, accessed on 26 January 2021) was implemented. In order to annotate the proteins, the KAAS’s online tool was used. These annotation results were mapped using the KEGG mapper against the KEGG database. According to the KEGG website, the putative pathways are organized hierarchically. Fisher’s exact test with two-tailed results was used to assess the statistical significance of the annotation, with a *p*-value < 0.05 regarded as significant.

### 2.8. Protein Domain Analysis

Based on the protein sequence alignment approach and the InterPro domain database (http://www.ebi.ac.uk/interpro/, accessed on 26 January 2021), Inter-ProScan (a sequence analysis application) annotated the functional domain description of the detected proteins. The enrichment of the DEPs versus all the detected proteins was examined using a two-tailed Fisher’s exact test after searching the InterPro database for each category of proteins. The protein domains were deemed significant if their *p*-value was less than 0.05.

### 2.9. Subcellular Localization

The subcellular location of the identified proteins was identified by the WoLF-PSORT-protein subcellular localization prediction tool. This tool predicts the subcellular location on the basis of amino acid residues of the protein. The program is an upgrade of the previous PSORT/PSORTII program. For the prokaryote species, the CELLO program was implemented to predict the subcellular location.

### 2.10. Enrichment-Based Clustering

To predict the hierarchical distribution of the identified protein based on the functions including GO, complex, pathways, and domain, all the categories, along with the *p*-values, were collated. The categories were narrowed down to ones that were enriched in at least one cluster with *p* < 0.05. This matrix of *p*-values was converted as the function x = −log10 (*p*-value). These x values were converted as the z function for each category. Finally, the z scores were clustered in genesis by a one-way hierarchical clustering. Using the “gplot” function of the R-package, cluster membership was depicted as a heatmap.

### 2.11. Protein–Protein Interaction Network

To gain an understanding of the protein–protein interaction, all the proteins identified were searched in the STRING database 10.5. The search was limited to proteins identified only and excluded external proteins. STRING results are presented as the confidence score to quantitatively define the protein–protein interaction; a confidence score > 0.7 was categorized as high confidence. Thus, we selected all interactions with a confidence score of seven, and the interaction network was visualized by the “networkD3” function of the R-package.

## 3. Results

### 3.1. Sample and Protein Identification

In this study, we have included two different sample types: the SARS-CoV-2 infection group (Q1_TCH, Q2_ZWB, Q3_WJH, Q5_JYJ, Q9_CT, and Q11_CCH) and healthy group (Q14_SZF, Q16_CJQ, Q17_CGS, Q20_BXX, Q32_LJM, and Q33_JYH). Mass spectrometry can identify a protein by determining its amino acid sequence. Mass spectrometry analysis includes two processes: primary mass spectrometry (MS1) and secondary mass spectrometry (MS2). The mass charge ratio (*m*/*z*) and peptide intensity (intensity) of the peptides eluted from HPLC MS1 were obtained by primary mass spectrometry; The secondary mass spectrometry determined the amino acid sequence of the peptide segment, and MS2 selected a single peptide segment to extract and split it to obtain the spectral data of the peptide segment. The retrieval software compares the secondary mass spectrum information with the corresponding database, combines the matching score and mismatch filtering to obtain the exact sequence of the peptide segment, and then splices the complete sequence of each protein to identify the protein. Quality control (QC) was achieved by performing a statistical analysis of the peptide length and average mass error PPM. The results suggest that the peptide length is ranged between 7aa and 23aa. The main peak was located at 11aa. The average mass error PPM has distributed between minus three and five, and the main peak was two. The above results indicate that quality control is qualified in this study (Supplementary Table S1). Subsequently, a total of 3319 peptides were identified in the secondary spectrum of the mass spectrometry after searching the theoretical data of proteins. Furthermore, we aligned the identified peptides to the corresponding proteins. The results show that a total of 891 proteins could be identified. It is notable that 639 proteins with multiple databases annotation of the 891 proteins can be quantified (Supplementary Table S1). Moreover, we tested the repeatability of the sample to reduce the experimental error. Principal component analysis (PCA), relative standard deviation (RSD), and Pearson’s correlation (Pearson’s correlation coefficient) were employed to evaluate the protein quantitative repeatability. Our results revealed that there was a strong positive correlation between the samples (0.62 and 0.84) (Supplementary Figure S1). RSD analysis indicated that the overall medium value of RSD was 0.05, which showed good repeatability between the two sets of samples (Supplementary Figure S2). PCA analysis, as shown in Supplementary Figure S3, suggested that most of the samples were clustered together. It was notable that Q1_TCH, Q2_ZWB, Q17_CGS, and Q20_BXX were away from the cluster center. This could be caused by individual differences or experimental errors.

### 3.2. DEPs and Functional Analysis

**Figure 1:**
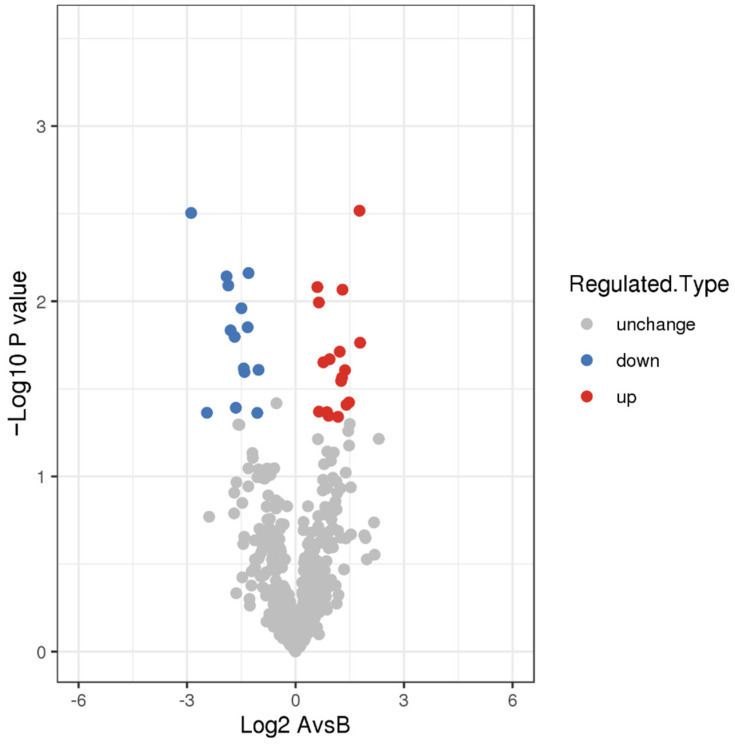
Volcano plot of DEPs. The blue dots represent proteins that are up-regulated (infection group vs. healthy group). The red dots represent down-regulated proteins (infection group vs. healthy group).

**Figure 2:**
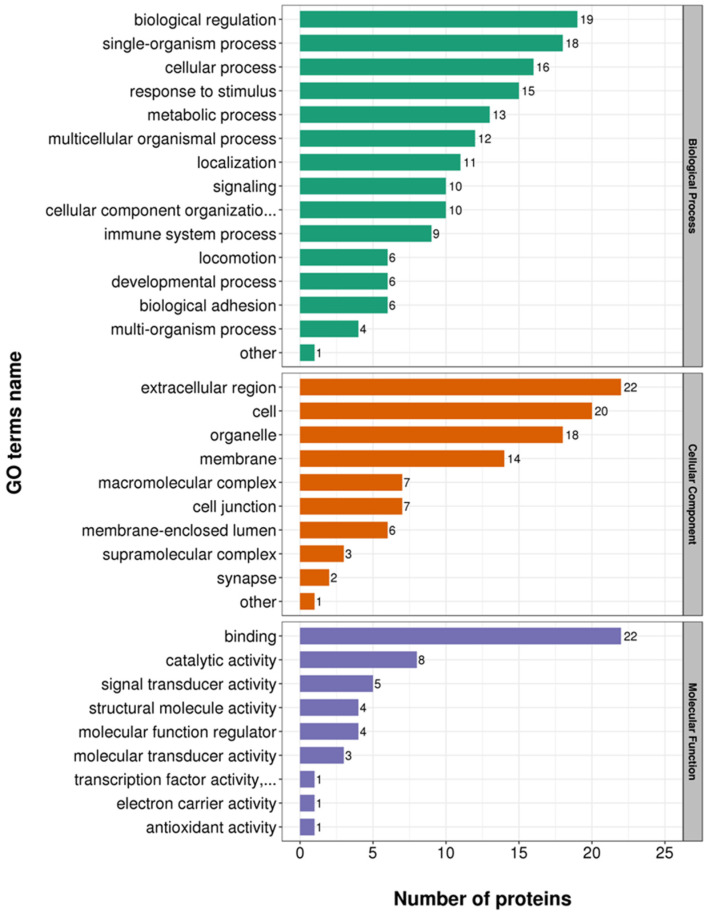
Statistical distribution chart of the 24 DEPsunder of each gene ontology (GO) category (second level). All GO results can be divided into three subgroups: cellular component, molecular function, and biological process.

**Figure 3:**
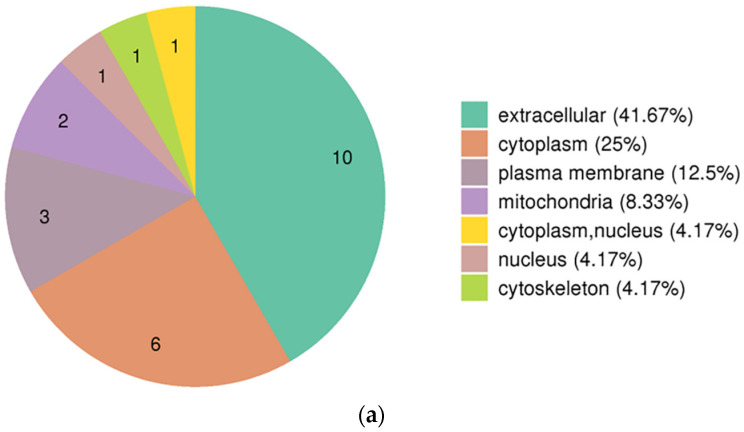

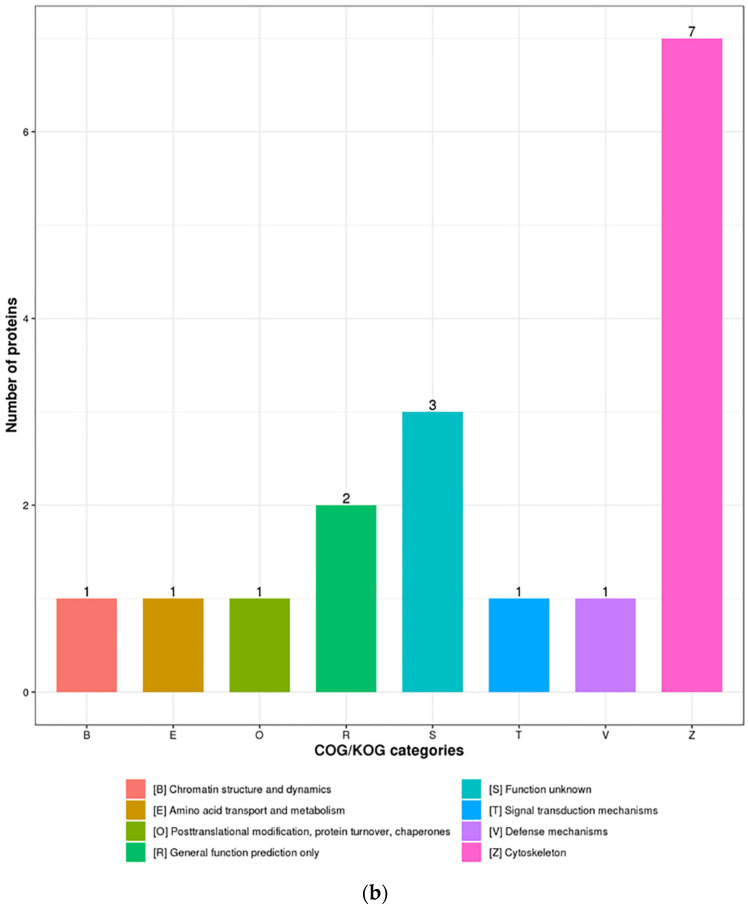
Subcellular localization and COG/KOG functional classification of the 24 DEPs. (**a**) Subcellular localization analysis of the 24 DEPs. (**b**) COG/KOG analysis of the 24 DEPs. Clusters of orthologous groups of proteins are named COG/KOG. The proteins that make up each COG/KOG are assumed to be derived from an ancestral protein. Therefore, the proteins in the same COG/KOG are considered orthologs or paralogs. Orthologs refer to proteins from different species that have evolved from vertical families (species formation), and characteristically preserve the same function as the original protein.

We compared the two datasets to identify differentially expressed proteins. We showed 24 proteins to be differentially expressed (infection group vs. healthy group) (Figure 1). Of these 24 genes, 10 genes were significantly up-regulated in the infection group compared with that in the healthy group, for example, IGLV3-19, IGLV3-1, CPXM1, CFD, APMAP, S100A9, HIST1H4A, PLTP, IL6R, and IGLV5-45. Meanwhile, the expression of 14 genes was significantly reduced in the infection group compared with that in the healthy group, for example, PCOLCE2, PDLIM1, SH3BGRL3, SH3BGRL, PIGR, PRNP, ACTB, POTEI, C4B, CDH5, TLN1, FLNA, MYL6B, and TPM3. In this study, statistical analysis was performed on the distribution of the 24 DEPs in the GO category (second level) (Figure 2 and Supplementary Table S2). From the perspective of biological processes, 15 different GO terms were allotted. Among these, the top t enriched terms were the cellular process, biological regulation, and single-organism process. In terms of the cellular component, ten GO terms were allotted. Among these terms, the extracellular region was the top significantly enriched term. From the molecular function perspective, nine GO terms were allotted. Out of these, binding was the most significantly over-represented term. Moreover, the subcellular localization and COG/KOG functional classification analysis of the DEPsshowed that these DEPs were primarily located in the extracellular, cytoplasm, and plasma membrane (Figure 3a and Supplementary Table S3). Notably, the COG/KOG functional classification analysis suggested that cytoskeleton (seven differentially expressed proteins), function unknown (three differentially expressed proteins), and general function prediction only (two differentially expressed proteins) were the top three classifications (Figure 3b and Supplementary Table S4).

### 3.3. Functional Enrichment Analysis of DEPs

**Figure 4:**
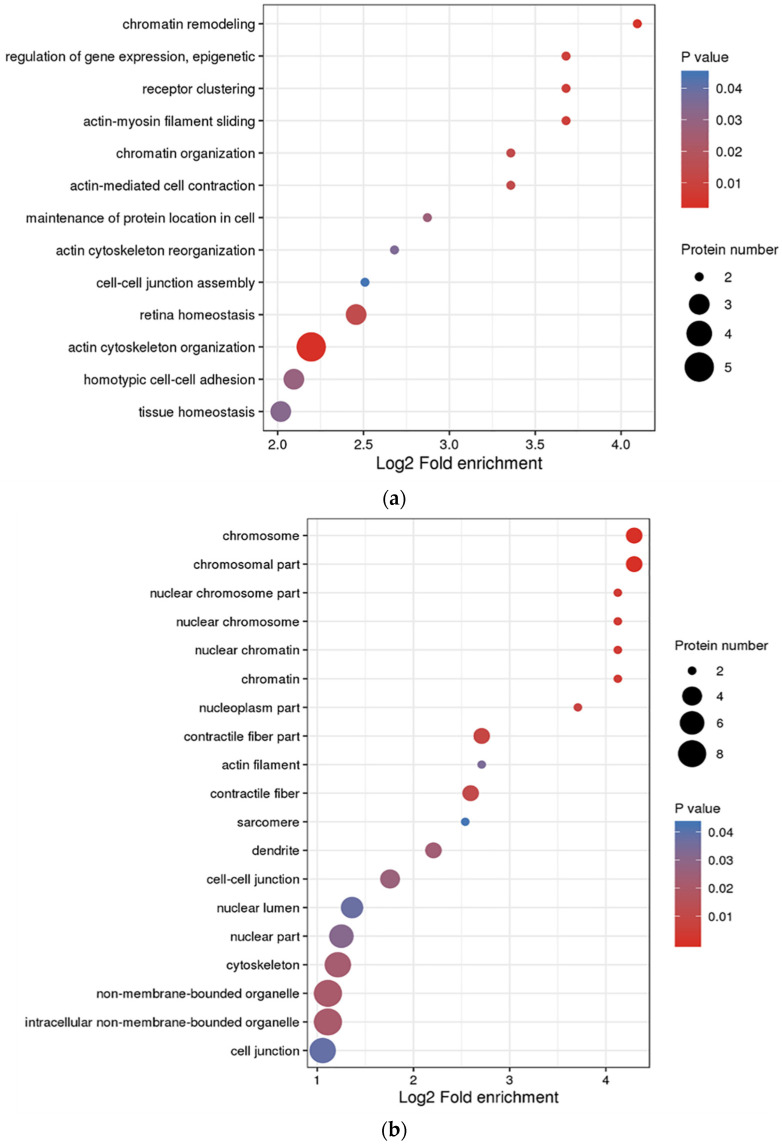

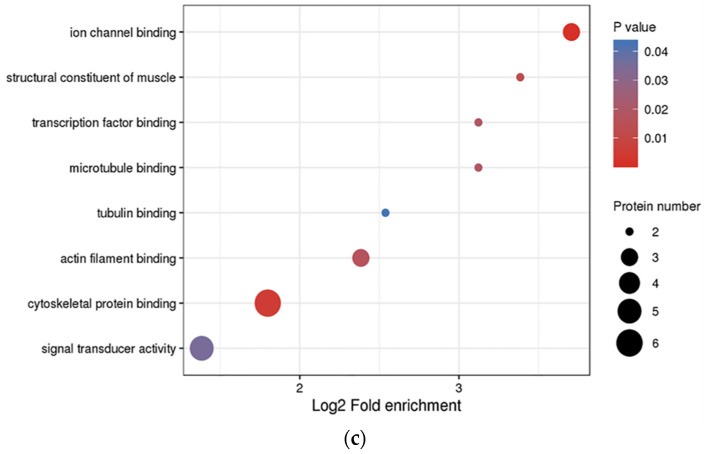
GO enrichment shown in the bubble plot of 24 DEPs in three categories: (**a**) biological process; (**b**) cellular component; (**c**) molecular function. For the enrichment test (Fisher’s exact test was used here), resuls show the most significant top 20 classifications. The *y*-axis denotes function classification, and the *x*-axis denotes the log2 converted value of the fold change. The significance of enrichment *p*-values is represented by colors. The circle size signifies the number of DEPs in each functional class or pathway.

**Figure 5:**
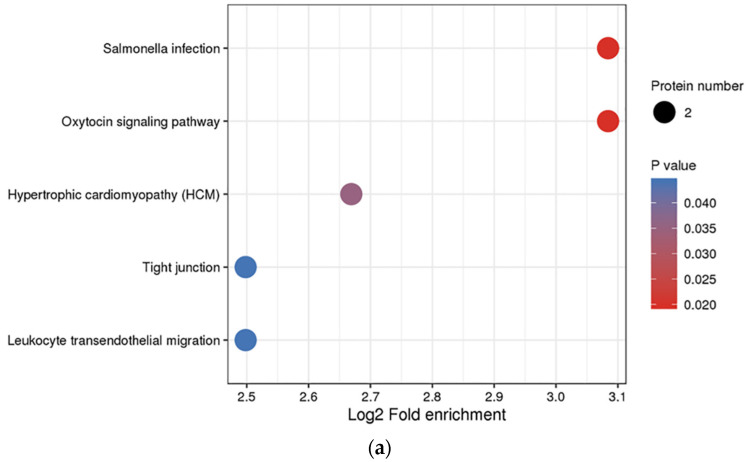

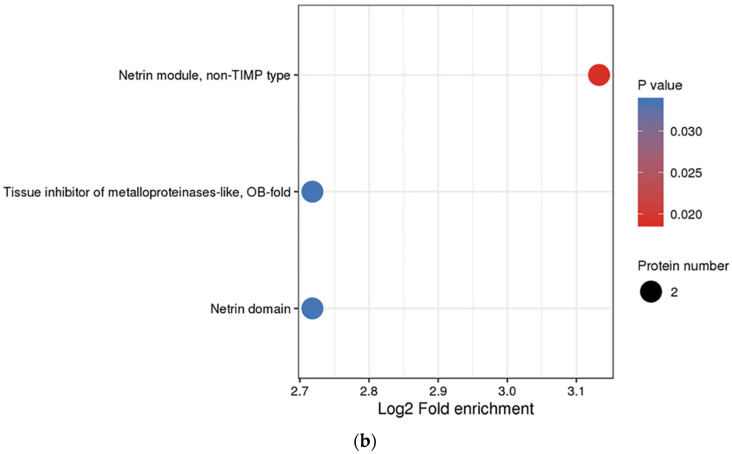
KEGG pathway and protein domain enrichment bubble plot of the 24 DEPs. (**a**) KEGG pathway enrichment analysis of the 24 DEPs. KEGG analysis provides information on molecular networks and their interaction, complexes, and biochemical reactions. (**b**) Repeatative component of proteins that appear regulary are know as protein domains, which generally include 25 to 500 amino acids in length. The significance of enrichment *p*-values is represented by colors. The circle size signifies the number of DEPs in each functional class or pathway.

With an aim to determine the function of DEPs, we performed the GO, KEGG, and protein domain enrichment analyses. Figure 4 demonstrates the results of the GO enrichment analysis on the 24 DEPs. In regard to biological processes, 13 GO terms were allotted. Among these terms, actin cytoskeleton organization (GO: 0030036), chromatin remodeling (GO: 0006338), and receptor clustering (GO: 0043113) were the first three significantly enriched terms. In regards to the cellular component, eight GO terms were assigned. Among these terms, the chromosomal part (GO: 0044427) and chromosome (GO: 0005694) were the most significantly enriched terms. In regard to the molecular function, eight GO terms were assigned; ion channel binding (GO: 0044325) was the most significantly over-represented term (Supplementary Table S5). Several biological functions were identified by incorporating various genes in vivo. The cellular signaling pathway enrichment analysis provided insight into the involvement of differentially expressed genes in signal transduction and biochemical pathways. Figure 5a (and Supplementary Table S6) demonstrated the involvement of five pathways with 24 significantly and differentially expressed proteins. It displayed that the oxytocin signaling pathway was the most enriched term. Two DEPs identified in our study seemed to be the participants in this pathway. Moreover, it is worth noting that the leukocyte transendothelial migration was also found to be significantly enriched. Furthermore, the protein domain enrichment analysis indicated that the netrin module (non-TIMP type) was the most enriched in the DEPs (Figure 5b and Supplementary Table S7). Notably, the immunoglobulin subtype was the most enriched in the DEPs that were down-regulated.

### 3.4. Protein–Protein Interaction Network

**Figure 6:**
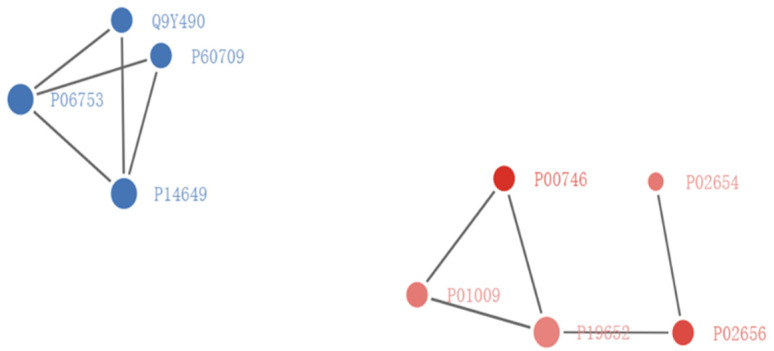
PPI network for DEPs interactions with three genes is extracted through STRING databse confidence cutoff of 0.900 (https://string-db.org/, accessed on 26 January 2021). The interaction network is reconstructed by using the Cytoscape software. The circles represent the differentially expressed proteins. Different colors represent the DEPs (blue is the down-regulated protein and red is the up-regulated protein). The size of the circle suggests the number of DEPs and their interacting proteins. In this study, we screened the top 50 proteins with the closest interaction and mapped the protein interaction network.

With an aim to further study the potential protein–protein interaction (PPI) between the 24 DEPs, the PPI network was explored. The results suggested that the up-regulated proteins and down-regulated proteins could be divided into two clusters (Figure 6). In the up-regulated proteins cluster, P00746 = CFD, P01009 = SERPINA1, P19652 = ORM2, P02656 = APOC3, and P02654 = APOC1 were clustered. Meanwhile, Q9Y490 = TLN1, P60709 = ACTB, P14649 = MYL6B, and P06753 = TPM3 were clustered in the down-regulated proteins. We speculate that both clusters could be important to the SARS-CoV-2 infection.

## 4. Discussion

At present, the novel coronavirus has spread to the whole world, causing a serious impact on global public health and the economy. Its appearance also made scientific researchers make unremitting efforts to fight against it with determination of daring to fight. The data shows that among COVID-19 patients, severe patients account for the majority of deaths, but the current clinical medical indicators are not observed in a timely manner, so early detection (prediction) and effective treatment of severe patients are crucial. The industry has found that there are many unique molecular changes in the serum of severe patients with COVID-19, and a series of biomarkers have been found, which are expected to provide guidance for predicting the development of mild patients to severe patients.

The rapid progress of proteomics provides a new approach to looking for serum molecular markers in patients with a viral infection. Tan et al. reported more than 20 proteins in the SARS-CoV genome [17]. Jiang et al. employed DIGE technology to analyze Vero-E6 cell lines infected with SARS-CoV for the first time and identified 355 DEPs [18]. Of these 355 proteins, 186 proteins were significantly and differentially expressed, which provided clues for understanding the SARS-CoV infection and pathogenic mechanisms [19]. Meanwhile, SILAC quantitative analysis of SARS-CoV-positive BHK21 cells showed that BAG3 restricts the replication of SARS-CoV [20]. Moreover, the analysis of the serum proteome of patients positive for SARS-CoV was helpful in discovering the biomarkers that can be used for the diagnosis, prognosis, and treatment [21]. Therefore, we have employed proteomics to probe the potential protein expression changes between SARS-CoV2-infected patients and healthy people in this study.

In the serum/plasma, the presence of high-abundance proteins, such as serum albumin, transferrin, binding globin, immunoglobulin, and lipoprotein, interferes with the identification of low-abundance biomarkers/proteins [22]. The concentration of different proteins in the serum varies greatly, and 22 of the main proteins account for 99% of the total serum protein. In human serum, it is estimated that there are more than 10,000 proteins, of which the major portion is found in low concentrations [23]. Therefore, reducing the complexity of serum samples, such as removing serum/plasma high-abundance proteins, is necessary to identify potential low-abundance disease-associated proteins. In this analysis, dyes, and protein A were used to remove high-abundance protein albumin in human serum samples. Additionally, we used Y3 ultrafiltration tubes to perform ultrafiltration centrifugation on the samples with an aim to effectively reduce the concentration of salt ions and lipids in the sample, thereby eliminating the effect of these interfering substances on isoelectric focusing.

The study identified a total of 24 DEPs. Among them, 10 protein expressions were increased, and on the other hand, 14 protein expressions were reduced. S100A9, an up-regulated protein in this analysis, is a member of the S100 calcium-binding protein family. The gene encoding the S100A9 is located at 1q21. The chromosome stability of this segment is poor, and various chromosome rearrangements easily occur. Previous reports suggest that this molecule can be involved in the final differentiation of epithelial cells [24]. S100A9 often forms a heterodimeric complex with S100A8 in a fold–fold symmetry resulting in the immunogenic protein-calprotectin [25]. The complex was initially known to be secreted by neutrophils. Subsequent studies have shown that the complex plays a crucial role in chronic and acute inflammation. The molecule was shown to participate in a variety of inflammatory reactions, which helps the host clear tumor and diseased cells [26]. PIGR, a down-regulated protein of the analysis, is an important component of mucosal immunity. This molecule is mainly distributed on the surface of the respiratory tract, gastrointestinal tract, and reproductive tract of the organisms. This molecule can be secreted into the mucosal cavity through endocytosis to form secreted immunoglobulin IgA, which can bind bacteria, viruses, parasites, and protoxins [27]. A study showed that the binding of the secreted immunoglobulin IgA to HIV surface capsid protein, which can hinder the adhesion between HIV and cells, subsequently halts intracellular viral replication [28]. Meanwhile, another study suggested that the secretory component can prevent the degradation of neutrophil elastase and further enhance the humoral immune effect of the respiratory tract, which is adapted to the virus infection [29].

The subunit C4B of complement C4 is also found to be down-regulated in virus-interfering serum. C4 is the main component of the classical pathway of complement. C4B preferentially combines with hydroxyl groups to form ester bonds, which is very important for the formation of complement immune complexes with soluble protein antigens [30]. Studies have shown that C4B genetic defects are associated with human immune complex diseases [31]. Aberations in the complement system are associated with diseases such as the dysfunction of the coagulation system [32]. Therefore, complement components are important regulators of inflammatory responses and immune responses. The reduction in serum C4B caused by SARS-CoV-2 infection needs to be studied in detail. Furthermore, IL6R protein expression was up-regulated in the infection group. IL-6 is a very important factor in protein synthesis, such as C-reactive protein. Similar to our results, another study has also shown that serum levels of IL-6 increase in acute and chronic inflammatory diseases [33]. Pathogens could stimulate endothelial cells and vascular components to produce IL-1β and/or TNF-α and induce the production of large amounts of cytokines, such as IL-6, which in turn causes the proteolysis of IL-6R. Subsequently, IL-6 and IL-6R drive the activation of the IL-6 transduction pathway in internal tissue cells by inhibiting leukocyte recruitment factors (CXCL1, CXCL8, and CX3CL1) and enhancing the activity of cytokines (CCL2, CCL8, CXCL5, and CXCL5) [34]. Therefore, IL-6 and IL-6R essentially play a role in controlling and alleviating acute neutrophil exudation in the human immune system.

Notably, IGLV3-19, IGLV3-1, and IGLV5-45 were also up-regulated during this analysis. These protein molecules originated from the immunoglobulin lambda light chain variable region, which is closely related to antigen–antibody reactions and drug target development.

## 5. Conclusions

In our analysis, we identified 24 DEPs signatures between the patients positive for SARS-CoV-2 and the healthy control. Ten DEPsin the SARS-CoV-2 infection group were found to be up-regulated, and 14 DEPs were down-regulated. The functional analysis of DEPs suggests that most of them are closely related to the human immune system. This study provides insight into the mechanism of the SARS-CoV-2 infection and paves a platform for future studies. The limitation of this study is the small sample size. Considering the high tautomerism of the human serum proteome, subsequent studies will further increase the number of subjects to improve the representativeness of the research results. In addition, Western Blotting will be used to confirm DEP in subsequent studies and to improve the accuracy of the study.

## Data Availability

Data associated with this study have been included in this pub-lished article. Additional files are available from the corresponding authors upon request.

## Supplementary Materials

The following supporting information can be downloaded at: https://www.mdpi.com/article/10.3390/tropicalmed7120397/s1, Table S1. Summary of the identified proteins in this study; Table S2. GO analysis of the 24 DEPs under each GO category (second level); Table S3. Subcellular location of the 24 DEPs; Table S4. COG/KOG analysis of the 24 DEPs; Table S5. GO enrichment analysis of the 24 DEPs; Table S6. KEGG enrichment analysis of the 24 DEPs; Table S7. Protein domain enrichment analysis of the 24 DEPs; Figure S1. Heatmap of Pearson correlation coefficients from all quantified proteins; Figure S2. Box plot of RSD (Relative Standard Deviation) distribution of repeated samples using quantified proteins; Figure S3. Two-dimensional scatter plot of PCA (principal component analysis) distribution of all samples using quantified.

## References

[1] World Health Organization (2020). Clinical Management of Severe Acute Respiratory Infection When Novel Coronavirus (nCoV) Infection Is Suspected: Interim Guidance.

[2] Ghelichi-Ghojogh M., Kalteh E.A., Fararooei M. (2020). Coronavirus disease 2019; epidemiology and recommendations. J. Prev. Epidemiol..

[3] Pal M., Berhanu G., Desalegn C., Kandi V. (2020). Severe Acute Respiratory Syndrome Coronavirus-2 (SARS-CoV-2): An Update. Cureus.

[4] Tu Y.F., Chien C.S., Yarmishyn A.A., Lin Y.Y., Chiou S.H. (2020). A Review of SARS-CoV-2 and the Ongoing Clinical Trials. Int. J. Mol. Sci..

[5] Jyl A., Zhi Y.A., Qw A., Zjz A., Ye Q.A., Rui L., Xyg A. (2020). The epidemic of 2019-novel-coronavirus (2019-nCoV) pneumonia and insights for emerging infectious diseases in the future. Microbes Infect..

[6] Rabaan A.A., Al-Ahmed S.H., Haque S., Sah R., Rodriguez-Morales A.J. (2020). SARS-CoV-2, SARS-CoV, and MERS-CoV: A comparative overview. Infez. Med..

[7] Long Q., Tang X., Shi Q., Li Q., Deng H., Yuan J., Hu J., Xu W., Zhang Y., Lv F. (2020). Clinical and immunological assessment of asymptomatic SARS-CoV-2 infections. Nat. Med..

[8] Li H., Liu S., Yu X., Tang S., Tang C. (2020). Coronavirus disease 2019 (COVID-19): Current status and future perspectives. Int. J. Antimicrob. Agents.

[9] Amirian E.S. (2020). Potential Fecal Transmission of SARS-CoV-2: Current Evidence and Implications for Public Health. Int. J. Infect. Dis..

[10] World Health Organization (2020). Coronavirus Disease (COVID-19) Situation Report.

[11] Geyer P.E., Voytik E., Treit P.V., Doll S., Kleinhempel A. (2019). Plasma Proteome Profiling to detect and avoid sample-related biases in biomarker studies. EMBO Mol. Med..

[12] Amiri-Dashatana N., Koushkib M., Abbaszadehc H.A. (2018). Proteomics Applications in Health: Biomarker and Drug Discovery and Food Industry. Iran. J. Pharm. Sci..

[13] Gutmann C., Takov K., Burnap S.A., Singh B., Ali H., Theofilatos K., Reed E., Hasman M., Nabeebaccus A., Fish M. (2021). SARS-CoV-2 RNAemia and proteomic trajectories inform prognostication in COVID-19 patients admitted to intensive care. Nat. Commun..

[14] Dong H., Li J., Lv Y., Zhou Y., Wang G., Hu S., He X., Yang P., Zhou Z., Xiang X. (2013). Comparative analysis of the alveolar macrophage proteome in ALI/ARDS patients between the exudative phase and recovery phase. BMC Immunol..

[15] Li Y., Meng Q., Wang L., Cui Y. (2021). TRIM27 protects against cardiac ischemia-reperfusion injury by suppression of apoptosis and inflammation via negatively regulating p53. Biochem. Biophys. Res. Commun..

[16] Ercan H., Schrottmaier W.C., Pirabe A., Schmuckenschlager A., Pereyra D., Santol J., Pawelka E., Traugott M.T., Schörgenhofer C., Seitz T. (2021). Platelet Phenotype Analysis of COVID-19 Patients Reveals Progressive Changes in the Activation of Integrin αIIbβ3, F13A1, the SARS-CoV-2 Target EIF4A1 and Annexin A5. Front. Cardiovasc. Med..

[17] Ghosh S., Parikh S., Nissa M.U., Acharjee A., Singh A., Patwa D., Makwana P., Athalye A., Barpanda A., Laloraya M. (2022). Semen Proteomics of COVID-19 Convalescent Men Reveals Disruption of Key Biological Pathways Relevant to Male Reproductive Function. ACS Omega.

[18] Dong X., Penrice-Randal R., Goldswain H., Prince T., Randle N., Donovan-Banfield I., Salguero F.J., Tree J., Vamos E., Nelson C. (2022). Analysis of SARS-CoV-2 known and novel subgenomic mRNAs in cell culture, animal model, and clinical samples using LeTRS, a bioinformatic tool to identify unique sequence identifiers. Gigascience.

[19] Tong T.R., Tabor E. (2006). Severe Acute Respiratory Syndrome Coronavirus (SARS-CoV). Perspectives in Medical Virology.

[20] Zhang L., Zhang Z., Zhang X., Lin F., Ge F. (2010). Quantitative Proteomics Analysis Reveals BAG3 as a Potential Target to Suppress Severe Acute Respiratory Syndrome Coronavirus Replication. J. Virol..

[21] Poon T.C., Pang R.T., Chan K.C., Lee N.L., Chiu R.W., Tong Y.K., Chim S.S., Ngai S.M., Sung J.J., Lo Y.M. (2012). Proteomic analysis reveals platelet factor 4 and beta-thromboglobulin as prognostic markers in severe acute respiratory syndrome. Electrophoresis.

[22] Luan J., Zhu X., Yu L., Li Y., He X., Chen L., Zhang Y. (2022). Construction of magnetic covalent organic frameworks functionalized by benzoboroxole for efficient enrichment of glycoproteins in the physiological environment. Talanta.

[23] Tu C., Rudnick P.A., Martinez M.Y., Cheek K.L., Stein S.E., Slebos R.J.C., Liebler D.C. (2010). Depletion of Abundant Plasma Proteins and Limitations of Plasma Proteomics. J. Proteome Res..

[24] Markowitz J., Carson W.E. (2013). Review of S100A9 biology and its role in cancer. Biochim. Biophys. Acta BBA—Rev. Cancer.

[25] Ehrchen J.M., Sunderkötter C., Foell D., Vogl T., Roth J. (2009). The endogenous Toll–like receptor 4 agonist S100A8/S100A9 (calprotectin) as innate amplifier of infection, autoimmunity, and cancer. J. Leukocyte Biol..

[26] Wang S., Song R., Wang Z., Jing Z., Wang S., Ma J. (2018). S100A8/A9 in Inflammation. Front. Immunol..

[27] Brandtzaeg P. (2013). Secretory IgA: Designed for Anti-Microbial Defense. Front. Immunol..

[28] Chojnacki J., Eggeling C. (2021). Super-Resolution STED Microscopy-Based Mobility Studies of the Viral Env Protein at HIV-1 Assembly Sites of Fully Infected T-Cells. Viruses.

[29] Newton A.H., Cardani A., Braciale T.J. (2016). The host immune response in respiratory virus infection: Balancing virus clearance and immunopathology. Semin. Immunopathol..

[30] Noris M., Remuzzi G. (2013). Overview of Complement Activation and Regulation. Semin. Nephrol..

[31] Soto K., Wu Y.L., Ortiz A., Aparício S.R., Yu C.Y. (2010). Familial C4B deficiency and immune complex glomerulonephritis. Clin. Immunol..

[32] Ricklin D., Reis E.S., Lambris J.D. (2016). Complement in disease: A defence system turning offensive. Nat. Rev. Nephrol..

[33] Tanaka T., Narazaki M., Kishimoto T. (2014). IL-6 in inflammation, immunity, and disease. Cold Spring Harb. Perspect. Biol..

[34] Narazaki M., Kishimoto T. (2018). The Two-Faced Cytokine IL-6 in Host Defense and Diseases. Int. J. Mol. Sci..

