# Supplementary material for: Screening and Analysis of Serum Protein Biomarkers Infected by Coronavirus Disease 2019 (COVID-19)
Source: Trop Med Infect Dis. 2022 Nov 25;7(12):397. doi: 10.3390/tropicalmed7120397 (PMC9788497; doi:10.3390/tropicalmed7120397)
Supplement: Supplementary file 1 [file tropicalmed-07-00397-s001.zip › Supplementary Figure S1-S3.pdf]

Supplementary Material

Summary of the identified proteins in this study: Repeatability

Pearson's correlation of quantitation												
	Q1_TCH	Q2_ZWB	Q3_WJH	Q5_JYJ	Q9_CT	Q11_CCH	Q14_SZF	Q16_CJQ	Q17_CGS	Q20_BXX	Q32_LJM	Q33_JYH
Q1_TCH	1.00	0.83	0.79	0.74	0.76	0.73	0.82	0.80	0.71	0.72	0.78	0.70
Q2_ZWB	0.83	1.00	0.82	0.71	0.78	0.76	0.82	0.78	0.73	0.70	0.79	0.68
Q3_WJH	0.79	0.82	1.00	0.71	0.82	0.84	0.82	0.83	0.77	0.74	0.80	0.68
Q5_JYJ	0.74	0.71	0.71	1.00	0.71	0.69	0.76	0.68	0.68	0.65	0.70	0.62
Q9_CT	0.76	0.78	0.82	0.71	1.00	0.82	0.80	0.79	0.78	0.78	0.81	0.65
Q11_CCH	0.73	0.76	0.84	0.69	0.82	1.00	0.75	0.78	0.78	0.75	0.81	0.71
Q14_SZF	0.82	0.82	0.82	0.76	0.80	0.75	1.00	0.82	0.77	0.76	0.80	0.68
Q16_CJQ	0.80	0.78	0.83	0.68	0.79	0.78	0.82	1.00	0.79	0.80	0.81	0.66
Q17_CGS	0.71	0.73	0.77	0.68	0.78	0.78	0.77	0.79	1.00	0.72	0.72	0.60
Q20_BXX	0.72	0.70	0.74	0.65	0.78	0.75	0.76	0.80	0.72	1.00	0.82	0.68
Q32_LJM	0.78	0.79	0.80	0.70	0.81	0.81	0.80	0.81	0.72	0.82	1.00	0.69
Q33_JYH	0.70	0.68	0.68	0.62	0.65	0.71	0.68	0.66	0.60	0.68	0.69	1.00

Figure S1. Heatmap of Pearson correlation coefficients from all quantified proteins.

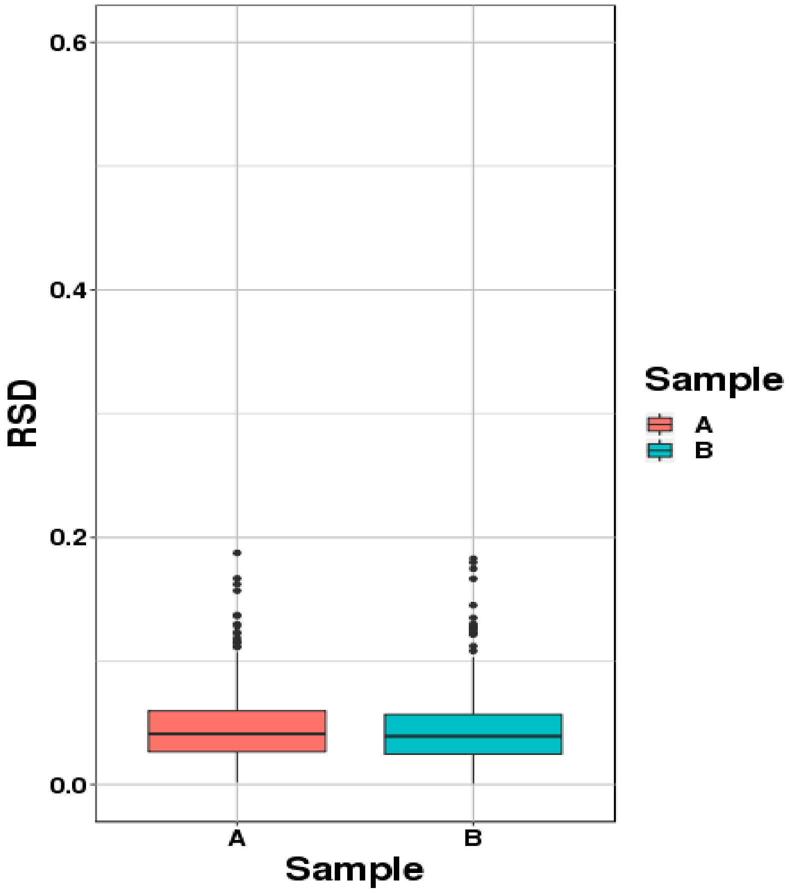

Figure S2. Box plot of RSD (Relative Standard Deviation) distribution of repeated samples using quantified proteins.

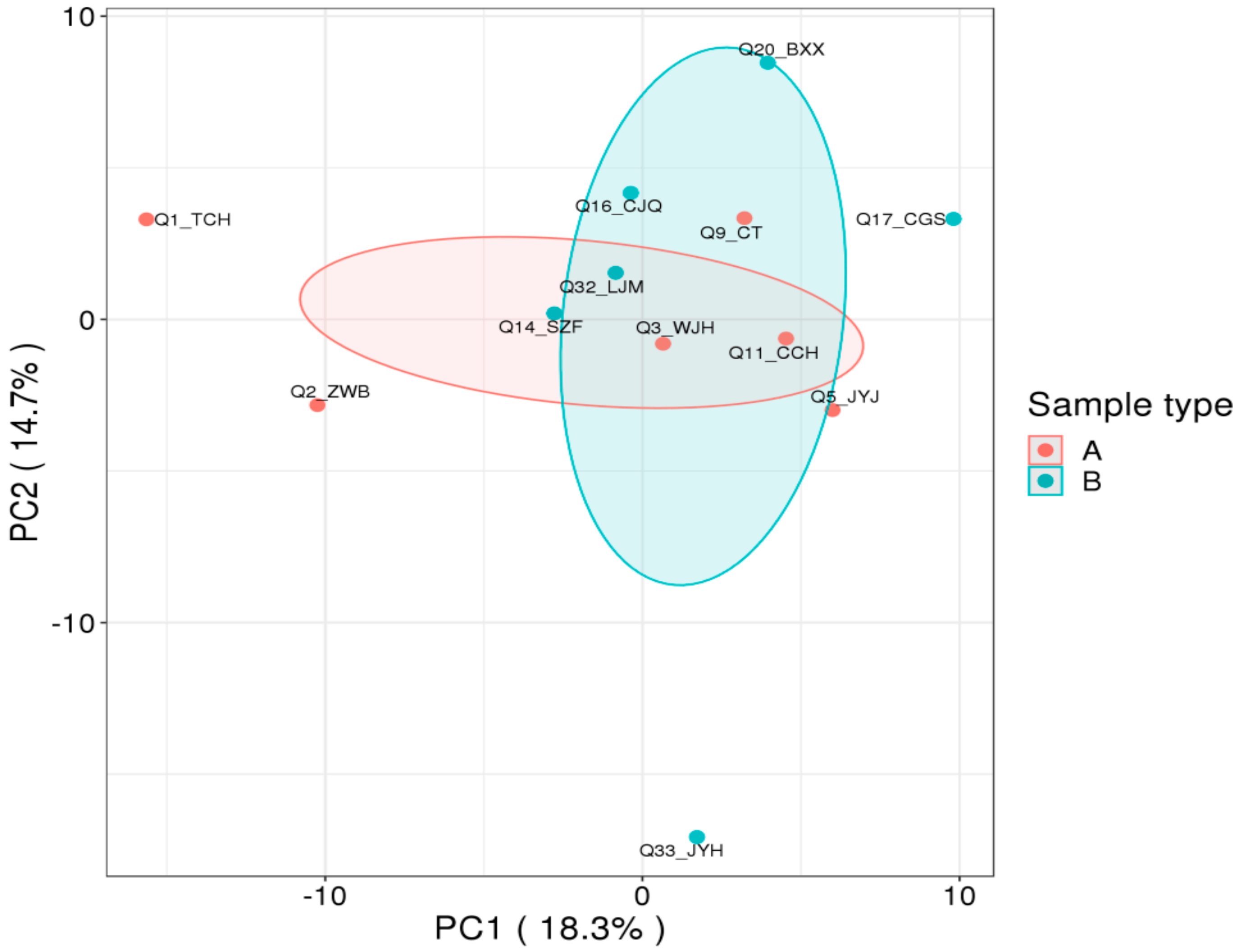

Figure 3. Two-dimensional scatter plot of PCA (principal component analysis) distribution of all samples using quantified proteins
.
